# Efficacy and safety of 0.05% cyclosporine ophthalmic emulsion in treatment of Chinese patients with moderate to severe dry eye disease: A 12-week, multicenter, randomized, double-masked, placebo-controlled phase III clinical study

**DOI:** 10.1097/MD.0000000000016710

**Published:** 2019-08-02

**Authors:** Di Chen, Shunhua Zhang, Ailing Bian, Jing Hong, Yingping Deng, Mingchang Zhang, Wei Chen, Yan Shao, Jialiang Zhao

**Affiliations:** a Department of Ophthalmology, Peking Union Medical College Hospital, Chinese Academy of Medical Sciences & Peking Union Medical College; b Department of Ophthalmology, Peking University Third Hospital, Beijing; c Department of Ophthalmology, West China Hospital, Sichuan University, Chengdu; d Department of Ophthalmology, Union Hospital, Tongji Medical College, Huazhong University of Science and Technology, Wuhan; e School of Ophthalmology and Optometry and Eye Hospital, Wenzhou Medical University, Zhejiang; f Department of Ophthalmology, Second Affiliated Hospital of Dalian Medical College, Dalian, China.

**Keywords:** clinical study, cyclosporine, dry eye disease, efficacy, safety

## Abstract

**Background:**

Dry eye disease (DED) is a chronic ocular surface disease that affects hundreds of millions of people worldwide. Although 0.05% cyclosporine ophthalmic emulsion (CsA OE) has long been prescribed in the U.S. for the treatment of DED, it is not commercially available in China. Our study aims to compare the efficacy and safety profile of 0.05% CsA OE versus vehicle in Chinese patients with moderate to severe DED.

**Methods:**

This was a multicenter, randomized, double-masked, 2-parallel-arm, 3-month phase III study. Patients with moderate to severe DED were randomized to receive twice-daily 0.05% CsA OE or its vehicle, along with unpreserved hypromellose eye drops 3 times per day. Patients were followed up at day 7, 28, 56, and 84, as well as 2 weeks after the medications were discontinued for safety assessment.

**Results:**

A total of 240 patients were randomized. The overall effective rate (OER) and efficacy index were significantly better in the CsA OE than vehicle group at all follow up times (all *P* < .05), and the OER of CsA OE and vehicle group at month 3 was 70.6% and 27.8%, respectively (*P* < .001) (primary endpoint). The patients in CsA OE group displayed a significant improvement in dry eye symptoms from day 28 and ocular surface test results from day 7 (all *P* < .05). The ocular surface disease index scores of 0.05% CsA OE treated patients were significantly better than those treated with vehicle control at day 56 and 84 (*P* = .0061 and <.001, respectively). Drug related adverse events (AEs) were recorded in 6(5%) and 3(2.5%) patients in the CsA OE and vehicle groups respectively (*P* = .4061) with ocular pain as the most frequently reported AEs, and it was mostly mild to moderate. There were no detrimental effects on visual acuity, intraocular pressure, or vital signs.

**Conclusions:**

Twice-daily instillation of 0.05% CsA OE was effective and well tolerated for the treatment of moderate to severe DED in Chinese population during the 3 months of the study.

## Abstract 2

Supplemental Digital Content is available in the text

## 1 Introduction

Dry eye disease (DED) is a chronic ocular surface disease that affects hundreds of millions of people throughout the world.^[1]^ The prevalence of DED ranges from 21% to 50.1% in Chinese population according to different studies.^[2–4]^ Moderate to severe DED is associated with significant pain, limitations in performing daily activities, reduced life quality, and often depression. It has been proved that ocular surface inflammation plays a vital role not only in the development of but as a downstream effect and propagator of DED.^[5]^ Since inflammation is such a key factor in the pathophysiology of DED, anti-inflammatory therapy has been recognized as an important component in the treatment regimen of DED.

Cyclosporine is an immunomodulatory drug with anti-inflammatory properties, and it has no serious adverse effects that are usually seen in corticosteroids treated patients. A phase III clinical trial in 2002 showed that both 0.05% and 0.1% cyclosporine ophthalmic emulsions (CsA OEs) were effective and well tolerated in treating moderate to severe DED in the U.S.^[6,7]^ Topical cyclosporine was approved by the FDA for the treatment of moderate to severe DED in 2003. Although 0.05% CsA OE has long been prescribed in the U.S., it is not commercially available in China and few studies have ever reported the efficacy and safety of 0.05% CsA OE in the Chinese population.^[8]^ Our study is a 12-week, multicenter, randomized, double-masked, placebo-controlled phase III clinical trial that aims to bring more evidence to the treatment of 0.05% CsA OE in moderate to severe Chinese DED patients.

## 2 Methods

The study was conducted in accordance with the Declaration of Helsinki and the Good Clinical Practice Guidelines. The study protocol and informed consent were reviewed and approved by the institutional review board of Peking Union Medical College Hospital before initiation (IRB#4790). Written informed consent was obtained from each patient before the start of the study, and power analysis was performed to justify the number of patients enrolled in the study.

### 2.1 Study design

This multicenter, randomized, double-masked, placebo-controlled parallel study compared 0.05% CsA OE (Sinqi Pharmaceutical, Shenyang, China) with vehicle over a 12-week treatment period in moderate to severe dry eye patients. All eligible patients received unpreserved hypromellose (HPMC) eye drops (Sinqi Pharmaceutical) as a basic treatment of dry eye. The study was conducted in 6 centers across China from November 2009 to October 2011.

Subjects recruited into the study were requested to discontinue use of any topical ophthalmic treatment (including their own artificial tears), and entered a 2-week washout period during which they administered 1 drop of unpreserved HPMC eye drops 3 times daily. After the washout period, eligible patients were randomized with an allocation ratio of 1:1 to receive either 0.05% CsA OE or its vehicle (1 drop, twice daily) along with HPMC eye drops (1 drop, 3 times daily) for 12 weeks. Subjects were randomized with the stratified random block design provided by Dr Bo Cao from Health Science Center of Peking University. Both the subjects and care providers were blinded to the intervention. Efficacy and safety were assessed at week 1 (day 7 ± 1 day), week 4 (day 28 ± 2 days), week 8 (day 56 ± 3 days), and week 12 (day 84 ± 3 days), as well as 14 ± 2 days after the medication was discontinued.

### 2.2 Participants

Inclusion criteria:

(1) aged 18 to 65 years old;
(2) visual acuity of study eye ≥2/20;
(3) moderate to severe DED.

Patients were required to have at least 2 symptoms of dry eye (eye dryness, burning, foreign body sensation, itching, stinging, visual fatigue, photophobia, and blurred vision). Each symptom had a severity score ranging from 0 (normal) to 4 (most severe). The selected eye must have a total symptom score ≥6. In the same eye (eligible eye), patients were also required to present at least 2 of the following 3 signs: tear film break-up time (TBUT)≤10 seconds, corneal fluorescein, and conjunctival lissamine green staining score (Oxford scheme)^[9]^ ≥5, Schirmer *I* test ≤5 mm/5 min. If only 1 eye of the patient met the inclusion criteria, then that eye was included in the study. If both eyes of the patient met the inclusion criteria, the eye with more severe symptoms was included. If the patient had same severity of dry eye symptoms in both eyes, then the right eye was included.

Exclusion criterion: history of ocular trauma, blepharitis, infection, or inflammation not associated with DED during the 3-month period preceding the screening visit; proptosis or eyelids cannot close; filamentous keratitis; ocular surgery within 3 months preceding the study; contact lens wearer; patients who were allergic to the medications used in this study; patients who had to use any systemic or local medications that might interfere with the study drug.

### 2.3 Efficacy assessment

Efficacy assessment included ocular symptoms, sign (eye redness), ocular surface tests, and ocular surface disease index (OSDI) score. Ocular symptoms included eye dryness, burning, foreign body sensation, itching, stinging, visual fatigue, photophobia, and blurred vision. Each symptom had a severity score ranging from 0 (normal) to 4 (extremely severe), with a total ocular symptom score from 0 to 32. Eye redness was also rated from 0 (normal) to 4 (extremely severe). Ocular surface tests included TBUT, Schirmer *I* test, corneal fluorescein, and conjunctival lissamine green staining (Oxford scheme). TBUT was scored as follows: 0, >10 seconds; 1, ≤10 and >5 seconds; 2, ≤5 and >2 seconds; 3, ≤2 and >0 second; 4, 0 second. Schirmer *I* test was scored as follows: 0, >10 mm/5 min; 1, ≤10 and >5 mm/5 min; 2, ≤5 and >2 mm/5 min; 3, ≤2 and >0 mm/5 min; 4, 0 mm/5 min.

Patients were evaluated and scored at the baseline and each visit based upon ocular symptoms, signs, and tests. OSDI was not included in the efficacy evaluation scoring system. While calculating the total ocular score, we set the weighted coefficients of objective parameters (TBUT, Schirmer *I* test, corneal, and conjunctival staining) as 3, subjective DED symptoms that are more frequently seen in DED patients (eye dryness, foreign body sensation, burning, visual fatigue) as 2 and subjective symptoms and signs that are less frequently seen in patients (itching, stinging, photophobia, blurred vision, and eye redness) as 1. The weighted score minimizes the influence of the subjective component on the primary endpoint. Efficacy index was defined as the percentage of improvement in total ocular score after medication compared with baseline at each visit. The overall effective rate (OER) was calculated as the percentage of the patients whose efficacy index was over 50% in total eligible cases.

The OER and efficacy index was set as the primary efficacy endpoint, and the changes of ocular surface symptoms, eye redness, dry eye test results, and OSDI score during the study were set as the secondary endpoint.

### 2.4 Safety assessment

Adverse events (AEs) were recorded throughout the study (all visits from baseline to month 3). AE was coded according to MedDRA19.1. Best corrected visual acuity (BCVA) and intraocular pressure (IOP) were also recorded at baseline and at months 3 to evaluate the safety profile of medications.

### 2.5 Local ocular tolerance assessments

Ocular symptoms related to drug instillation were assessed by asking the patient whether he or she felt any ocular discomfort within 10 minutes after instillation of the eye drop. If the answer was “no,” then the score was recorded as 0. If the answer was “yes,” the patient graded its severity on a 3-point scale (1, mild; 2, moderate; or 3, severe).

### 2.6 Sample size

The sample size calculation was based on an estimated OER of 70% of the study group and 40% of control group with a 2-sided *t* test at 5% significance level and at 80% power. A sample size of 31 patients per group was deemed necessary in order to detect a clinically relevant change. According to certain provisions of *Administration of Drug Registration in China*, 100 patients per group were required to meet the regulations. Thus, a total sample size of 240 patients (120 per group) was to be recruited into study with an anticipated dropout rate of 20%.

### 2.7 Statistical analysis

SAS 9.3 was applied for the statistical analysis. The full analysis set (FAS) was used for the baseline and efficacy evaluation. Missing data for the primary efficacy variables were imputed using the last observation carried forward method. The per protocol set (PPS) population included patients from the FAS who did not have any major protocol deviations. The efficacy outcomes were analyzed for both the FAS and PPS datasets. Safety set (SS) included all the patients who instilled the study drug and had safety data recorded at least once.

The data were given as mean ± standard deviation and compared with Student *t* test for normally distributed data. For the data that were not normally distributed, the data were displayed as median (range) and compared with the Wilcoxon rank-sum test. Chi-square test or Fisher exact test was used for the analysis of categorical data. CMH chi-square test was applied for the analysis of ordinal category data. *P* < .05 was considered statistically significant.

## 3 Results

**Figure 1:**
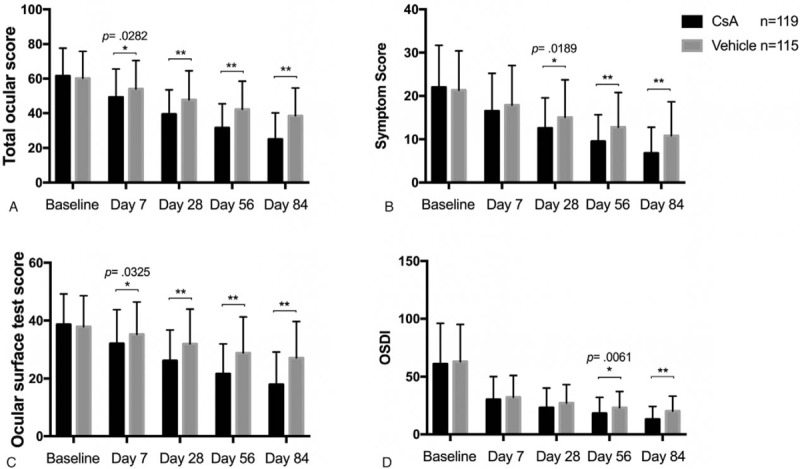
Comparison of the secondary endpoints within 84 days of randomized treatment with 0.05% CsA OE and vehicle controls in patients with moderate to severe dry eye disease. Values of total ocular score (A), symptom score (B), ocular surface test score (C) and OSDI (D) were displayed respectively. ^∗^*P* < .05, ^∗∗^*P* < .001, 2-tailed *t* test. CsA OE = cyclosporine ophthalmic emulsion, OSDI = ocular surface disease index.

Among the 240 patients who were recruited, 219 completed the study and the dropout rate was 8.75% (21/240). A total of 234 patients, 119 in the CsA OE group and 115 in the vehicle group, were included in the FAS. A total of 177 patients, 88 in the CsA OE group and 89 in the vehicle group, were included in the PPS. Of the 57 patients that were not included in the PPS population, 41 were due to violation of the rules on combined use of drugs (4 were suspended due to the use of prohibited drugs during the study and 37 were traced at the end of study), 20 (12 in the CsA OE group and 8 in the vehicle group) were excluded due to loss of follow-up or study suspension (Supplementary Table 1). All of the 240 patients had safety assessment and were included in the SS. In general, patients showed good compliance in this study with an overall compliance rate of 98.3% in the CsA OE group and 100% in the vehicle group (*P* = .4979). Demographic (age and sex) and baseline disease characteristics were generally well balanced across the randomized treatment groups (Table 1). The patients were randomly assigned with the treatments and showed similar baseline symptom and test scores between 2 groups (Fig. 1).

**Table 1:**
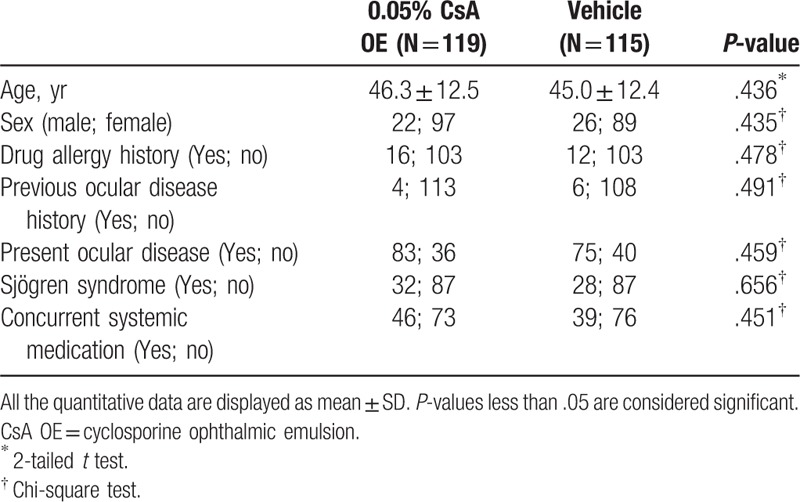
Demographics and baseline characteristics in the full analysis set.

### 3.1 Efficacy results

Unless otherwise specified, all efficacy results presented here were assessed in the FAS population, and either confirmed or supported by analyses performed in the PPS population.

#### 3.1.1 Primary efficacy endpoint

The OER of CsA OE group and vehicle group at month 3 was 70.6% and 27.8% (*P* < .001), and the difference between 2 groups was 42.8% (95% confidence interval [31.2%–54.3%]) (Table 2). According to the Breslow test, the *P* values were all >.38, and no significant inconsistency was found between the centers. After the correction of the central effect, the difference was statistically significant (*P* < .001). The OER and efficacy index of CsA OE were significantly higher than those of the vehicle at all follow-up times, starting from 1 week after medication (Table 2).

**Table 2:**
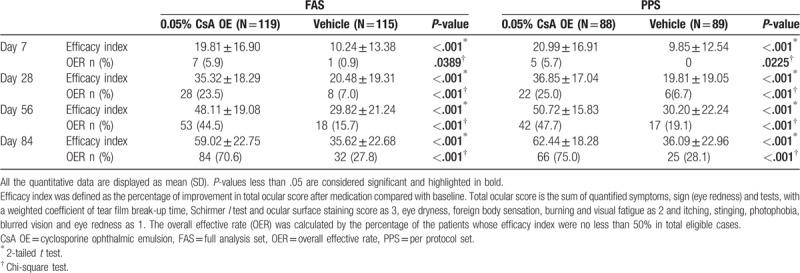
Comparison of primary endpoints between 0.05% CsA OE group and vehicle group at different time points during the study in the FAS and PPS.

#### 3.1.2 Secondary efficacy endpoint

The patients in CsA OE group showed significant less total ocular score from day 7, less symptom score from day 28 and less ocular surface test score from day 7 (Fig. 1). The difference between CsA OE and vehicle group was more obvious with treatment time in total ocular score, symptom score, and ocular surface test score (Fig. 1). Specifically speaking, patients in CsA OE group reported significant less eye dryness, burning, foreign body sensation, stinging, visual fatigue, and photophobia at day 56 and 84 (all *P* < .05, Supplementary Table 2–5, 7, and 8). There were no significant differences in itching, blurred vision and eye redness between 2 groups at all visits (all *P* > .05, Supplementary Table 6, 9, and 10). TBUT, Schirmer *I* test and ocular surface staining (Oxford scheme) in the CsA OE group had significant improvement compared with those in the control from day 28 to the end of study (Table 3, Supplementary Table 11–13). All these results of secondary efficacy endpoint were consistent with that of primary endpoint, indicating CsA OE was effective in treating patients with moderate or severe dry eye compared with vehicle control.

**Table 3:**
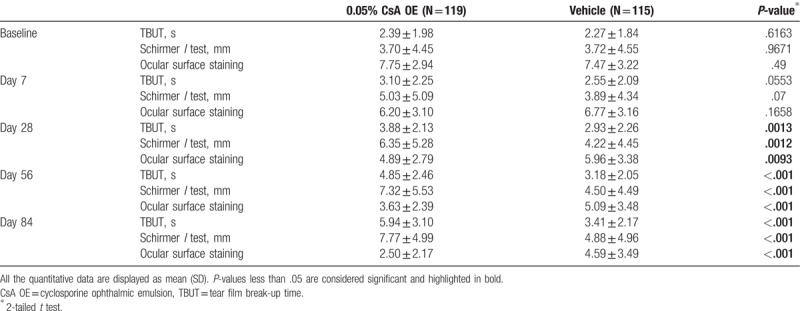
Comparison of tear film break-up time, Schirmer *I* test and ocular surface staining score (Oxford scheme) between 0.05% CsA OE group and vehicle group at different time points during the study in the full analysis set.

Since OSDI is a subjective self-assessment, it was not included in the efficacy evaluation system and listed as another indicator. The OSDI scores of CsA OE treated patients were significantly better than those treated with vehicle control at day 56 and 84 (*P* = .0061 and <.001, respectively) (Fig. 1).

### 3.2 Adverse events

Total AEs were reported in 15 (12.5%) and 11 (9.2%) patients in the CsA OE and vehicle groups, respectively (*P* = .4061, Table 4). Drug related AEs were recorded in 6 (5%) and 3 (2.5%) patients in the CsA OE and vehicle groups respectively (*P* = .4061) with ocular pain as the most frequently reported AEs. Four cases reported moderate ocular pain in the CsA OE group, 2 cases reported mild, and another 2 reported severe ocular pain in the vehicle group. Cornea staining, swollen eyelid, and tearing occurred in 1 case in CsA OE group. No severe AE was reported during the study. There were no changes in BCVA or IOP over the course of the study (data not shown).

**Table 4:**
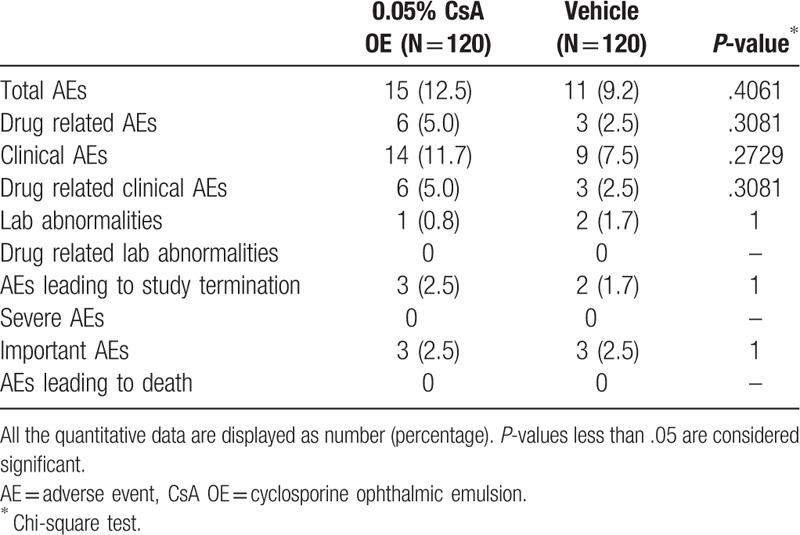
Comparison of AEs between 0.05% CsA OE group and vehicle group in the safety set.

### 3.3 Local ocular tolerance

The percentage of patients experiencing ocular discomfort related to eye drop instillation decreased in both treatment groups between baseline (18.3% for CsA OE and 10.5% for vehicle) and day 84 (4.6% for CsA OE and 5.4% for vehicle), with no significant difference between 2 groups at all visits (Table 5). In addition, the majority of patients experienced mild and transient (≤10 minutes) ocular discomfort after drug instillation.

**Table 5:**
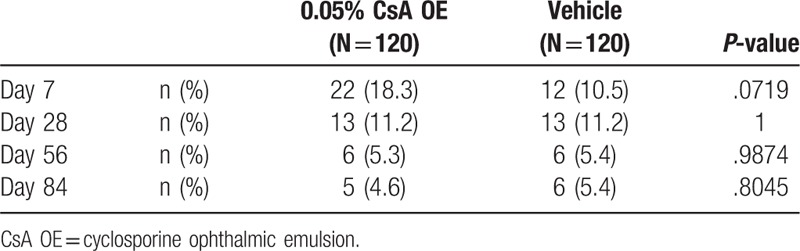
Comparison of the number of patients who reported ocular discomfort within 10 min after eye drop instillation between 0.05% CsA OE and vehicle group.

## 4 Discussion

The primary objective of this study was to demonstrate the efficacy and safety of CsA OE in Chinese patients with moderate to severe DED. The OER and efficacy index were significantly better in the CsA OE group than the vehicle at all follow-up times. These results demonstrated the superiority of CsA OE over vehicle in treating moderate to severe DED and were consistent with the findings of other studies.^[7,8,10–12]^ As indicated by the TFOS DEWS II Pathophysiology Report, inflammatory responses triggered by tear hyperosmolarity lead to chronic ocular surface damage in DED.^[5]^ Anti-inflammatory treatment is a more reasonable approach besides traditional symptomatic treatment of dry eye. CsA has long been proven its anti-inflammatory effects through multiple mechanisms, including the inhibition of T lymphocyte activation and the consequent cytokine production,^[13,14]^ the blocking of epithelium apoptosis marker expression,^[15]^ the increase of conjunctival goblet cell density and the decrease of squamous metaplasia.^[16]^ Due to the time-taking effects of CsA, the benefits of CsA usually begin after 1 month of treatment and a therapy of at least 3 months seems to be indispensable. Our study also showed a significant improvement of ocular symptoms in the CsA OE group after 1 month of treatment and the difference between CsA OE and vehicle was more obvious at month 3.

The significant improvement of dry eye tests in CsA OE group was observed nearly 1 month earlier than the symptoms in our study. This phenomenon is possibly due to the relatively higher sensitivity of tests than symptoms in the DED diagnosis.^[17]^ It could also be attributed to the treatment used in the control group. Considering the hydrophobicity and low aqueous solubility of CsA, ophthalmic emulsion was used as the vehicle in the ocular surface delivery for CsA in our study.^[18]^ Patients in the control group received both emulsion and HPMC eye drops as a basic treatment of DED, which might alleviate the symptoms and thus delay the finding of significance. This finding also shows the well-documented weak correlation between tests and symptoms in DED.^[17,19,20]^

Since dry eye tests are generally more specified than the symptoms in diagnosing DED, we set the weighted coefficient of test scores as 3 when calculating the total ocular score.^[21,22]^ We also set the weighted coefficient of more-reported dry eye symptoms in Chinese patients (eye dryness, foreign body sensation, burning, and visual fatigue) as 2 and others (itching, stinging, photophobia and blurred vision) as 1 in the analysis.^[4,22]^ Thus, the total ocular score of patients in the CsA OE group was significantly better than controls after 1 week of treatment, while it took 1 or 2 months for each individual symptom or test to have such a significant improvement. Chen et al. also compared the efficacy of 0.05% CsA OE with vehicle in treating moderate to severe DED in the Chinese population.^[8]^ However, they did not find a significant difference in burning, photophobia or TBUT, which was possibly because of the relatively shorter length of their study (8 weeks). Unlike previous study by Sall et al, no significant differences in blurred vision or itching were found in our study.^[7]^ This is probably because these 2 symptoms are relatively less reported by dry eye patients in the Chinese population.

Although the most common clinical sign that is suggestive of ocular surface inflammation is conjunctival redness, both Chen et al's study and our study did not find a significant difference in eye redness between 2 groups.^[8,17]^ Both 2 studies applied a relatively subjective grading system for the evaluation of eye redness based upon investigator's experience. A more objective and quantitative documentation methods using digital imaging analysis might be adopted for future studies to explore the subtle changes of conjunctival erythema.^[23,24]^ Besides, eye redness could be caused by any stimulus to the ocular surface, not just DED. It was also reported that CsA OE could induce conjunctival erythema.^[7]^ Thus a more specific indicator of ocular surface inflammation should be applied to evaluate the anti-inflammatory effect of CsA OE.

OSDI is the most widely used questionnaire for DED clinical trials. ^[25]^The OSDI measures frequency of experiencing symptoms, environmental triggers, and vision-related life quality.^[26]^ We included OSDI score as a supplement to Chen et al's study. The OSDI scores of both groups in our study had an obvious decrease after treatment, approaching to 0 at month 3. Patients in CsA OE group had more improvements in OSDI after month 2, and the statistically significant differences in OSDI between the groups strongly support that CsA OE relieves the uncomfortable ocular symptoms associated with DED and improves the quality of life.

Our study proved the safety of CsA OE, which was well tolerated in most patients with findings consistent with the expected safety profile of CsA. There were no detrimental effects on visual acuity, IOP, or vital signs.

Our study also has some limitations. First, since DED is a chronic disease that requires continuing treatment, the length of our study might not be long enough to prove the long-term effects of CsA on dry eye. Second, a more objective indicator of ocular surface inflammation might be needed to test the anti-inflammatory effects of CsA in dry eye patients.

In conclusion, twice-daily instillation of 0.05% CsA OE was well tolerated and effective for the treatment of moderate to severe DED in Chinese population during the 3 months of the study, with significant higher efficacy index and OER observed from as early as week 1.

## Acknowledgments

We appreciate Jianfeng Luo from Fudan University and Bo Cao from Health Science Center of Peking University for their help in the statistical analysis.

## Author contributions

**Conceptualization:** Shunhua Zhang.

**Data curation:** Di Chen, Shunhua Zhang, Ailing Bian, Jing Hong, Yingping Deng, Mingchang Zhang, Wei Chen, Yan Shao.

**Formal analysis:** Di Chen, Shunhua Zhang.

**Funding acquisition:** Shunhua Zhang, Jialiang Zhao.

**Investigation:** Di Chen, Ailing Bian, Yingping Deng, Mingchang Zhang, Wei Chen, Yan Shao.

**Methodology:** Ailing Bian.

**Project administration:** Shunhua Zhang.

**Resources:** Jing Hong, Jialiang Zhao.

**Software:** Di Chen.

**Supervision:** Jing Hong, Yingping Deng, Mingchang Zhang, Wei Chen, Yan Shao, Jialiang Zhao.

**Validation:** Jing Hong, Jialiang Zhao.

**Writing – original draft:** Di Chen.

**Writing – review and editing:** Di Chen, Shunhua Zhang.

Shunhua Zhang orcid: 0000-0002-5093-8369.

## Supplementary Material

Supplemental Digital Content
