# Supplementary material for: Efficacy and safety of 0.05% cyclosporine ophthalmic emulsion in treatment of Chinese patients with moderate to severe dry eye disease: A 12-week, multicenter, randomized, double-masked, placebo-controlled phase III clinical study
Source: Medicine (Baltimore). 2019 Aug 2;98(31):e16710. doi: 10.1097/MD.0000000000016710 (PMC6709181; doi:10.1097/MD.0000000000016710)
Supplement: Supplemental Digital Content [file medi-98-e16710-s001.docx]

Table 1. Details of the patients who lost follow-up or were suspended in the full analysis set

|  | 0.05% CsA OE  (N=119） | Vehicle (N=115) |
| --- | --- | --- |
| Loss of follow-up or study suspension | 12 (10%) | 8 (6.7%) |
| Suspended by investigator | 3 (2.5%) | 2 (1.7%) |
| Suspended by investigator due to allergy or AE | 1 (0.8%) | 2 (1.7%) |
| Suspended by investigator due to other diseases | 2 (1.7%) | 0 |
| Loss of follow-up | 9 (7.5%) | 6 (5.0%) |
| Loss of follow-up  Day 7-28 | 2 (1.7%) | 2 (1.7%) |
| Loss of follow-up  Day 29-56 | 3 (2.5%) | 2 (1.7%) |
| Loss of follow-up  Day 57-84 | 4 (3.4%) | 2 (1.7%) |
| Violation of the rules on combined use of drugs | 19 (15.8%) | 18 (15.0%) |

All the data were presented as number (percentage).

CsA OE: cyclosporine ophthalmic emulsion

AE: adverse effect

Table 2. Comparison of eye dryness score between 0.05% cyclosporine ophthalmic emulsion (CsA OE) group and vehicle group at different time points during the study in the full analysis set

|  | 0.05%  CsA OE  (N=119） | Vehicle (N=115) | Statistics^§^  (CMH) | *p* value |
| --- | --- | --- | --- | --- |
| Baseline n (%)  0 - normal  1 - mild  2 - moderate  3 - severe  4 - extremely severe | 0  7(5.9%)  30 (25.2 %)  47 (39.5 %)  35 (29.4 %) | 0  11(9.6%)  33 (28.7 %)  44 (38.3 %)  27 (23.5 %) | 2.0862 | .5547 |
| Day 7 n (%)  0 - normal  1 - mild  2 - moderate  3 - severe  4 - extremely severe | 2(1.7%)  22 (18.5 %)  46 (38.7 %)  41 (34.5 %)  8(6.7%) | 1(0.9%)  23(20.2 %)  43 (37.7 %)  34 (29.8 %)  13 (11.4 %) | 2.1848 | .7018 |
| Day 28 n (%)  0 - normal  1 - mild  2 - moderate  3 - severe  4 - extremely severe | 8(7.0%)  37 (32.2 %)  45 (39.1 %)  25 (21.7 %)  0 | 2(1.8%)  35 (31.3 %)  46 (41.1 %)  23 (20.5 %)  6(5.4%) | 9.6691 | **.0464** |
| Day 56 n (%)  0 - normal  1 - mild  2 - moderate  3 - severe  4 - extremely severe | 12 (10.7 %)  63 (56.3 %)  29 (25.9 %) 8(7.1%)  0 | 3(2.8%)  48 (44.4 %)  41 (38.0 %)  13 (12.0 %)  3(2.8%) | 13.545 | **.0089** |
| Day 84 n (%)  0 - normal  1 - mild  2 - moderate  3 - severe  4 - extremely severe | 27 (25.2 %)  67 (62.6 %)  11 (10.3 %) 2(1.9%)  0 | 10(9.3%)  49 (45.4 %)  38 (35.2 %)  9(8.3%)  2(1.9%) | 31.784 | **< .001** |

^§^ CMH chi-square test;

*p*-values less than 0.05 are considered significant and highlighted in bold.

CsA OE: cyclosporine ophthalmic emulsion

Table 3. Comparison of foreign body sensation score between 0.05% cyclosporine ophthalmic emulsion (CsA OE) group and vehicle group at different time points during the study in the full analysis set

|  | 0.05%  CsA OE  (N=119） | Vehicle  (N=115) | Statistics^§^  (CMH) | *p* value |
| --- | --- | --- | --- | --- |
| Baseline n (%)  0 - normal  1 - mild  2 - moderate  3 - severe  4 - extremely severe | 11(9.2%)  26 (21.8 %)  38 (31.9 %)  26 (21.8 %)  18 (15.1 %) | 17 (14.8 %)  26 (22.6 %)  36 (31.3 %)  24 (20.9 %)  12 (10.4 %) | 2.5412 | .6373 |
| Day 7 n (%)  0 - normal  1 - mild  2 - moderate  3 - severe  4 - extremely severe | 28 (23.5 %)  37 (31.1 %)  33 (27.7 %)  16 (13.4 %)  5(4.2%) | 21 (18.4 %)  36 (31.6 %)  32 (28.1 %)  17 (14.9 %)  8 (7.0 %) | 1.6381 | .8019 |
| Day 28 n (%)  0 - normal  1 - mild  2 - moderate  3 - severe  4 - extremely severe | 42 (37.5 %)  57 (50.9 %)  10(8.9%)  3(2.7%)  0 | 29 (26.9 %)  41 (38.0 %)  27 (25.0 %)  9(8.3%)  2(1.9%) | 6.9392 | .1391 |
| Day 56 n (%)  0 - normal  1 - mild  2 - moderate  3 - severe  4 - extremely severe | 63 (56.3 %)  39 (34.8 %)  10 (8.9 %)  0  0 | 56 (51.9 %)  27 (25.0 %)  18 (16.7 %)  7 (6.5 %)  0 | 17.656 | **.0014** |
| Day 84 n (%)  0 - normal  1 - mild  2 - moderate  3 - severe  4 - extremely severe | 59 (55.1 %)  42 (39.3 %) 6(5.6%)  0  0 | 36 (33.3 %)  45 (41.7 %)  21 (19.4 %)  4(3.7%)  2(1.9%) | 19.908 | **<0.001** |

^§^ CMH chi-square test;

*p*-values less than 0.05 are considered significant and highlighted in bold.

CsA OE: cyclosporine ophthalmic emulsion

Table 4. Comparison of burning score between 0.05% cyclosporine ophthalmic emulsion (CsA OE) group and vehicle group at different time points during the study in the full analysis set

|  | 0.05%  CsA OE  (N=119） | Vehicle (N=115) | Statistics^§^  (CMH) | *p* value |
| --- | --- | --- | --- | --- |
| Baseline n (%)  0 - normal  1 - mild  2 - moderate  3 - severe  4 - extremely severe | 32 (26.9 %)  40 (33.6 %)  25 (21.0 %)  13 (10.9 %)  9 (7.6 %) | 36 (31.3 %)  32 (27.8 %)  24 (20.9 %)  16 (13.9 %)  7 (6.1 %) | 1.63 | .8034 |
| Day 7 n (%)  0 - normal  1 - mild  2 - moderate  3 - severe  4 - extremely severe | 42 (35.3 %)  43 (36.1 %)  24 (20.2 %)  7 (5.9 %)  3 (2.5 %) | 38 (33.3 %)  39 (34.2 %)  23 (20.2 %)  10 (8.8 %)  4 (3.5 %) | 0.9776 | .9132 |
| Day 28 n (%)  0 - normal  1 - mild  2 - moderate  3 - severe  4 - extremely severe | 49 (42.6 %)  46 (40.0 %)  17 (14.8 %)  3 (2.6 %)  0 | 44 (39.3 %)  40 (35.7 %)  20 (17.9 %)  6 (5.4 %)  2 (1.8 %) | 3.8746 | .4232 |
| Day 56 n (%)  0 - normal  1 - mild  2 - moderate  3 - severe  4 - extremely severe | 63 (56.3 %)  39 (34.8 %)  10 (8.9 %)  0  0 | 56 (51.9 %)  27 (25.0 %)  18 (16.7 %)  7 (6.5 %)  0 | 11.757 | **.0083** |
| Day 84 n (%)  0 - normal  1 - mild  2 - moderate  3 - severe  4 - extremely severe | 77 (72.0 %)  25 (23.4 %)  4 (3.7 %)  1 (0.9 %)  0 | 59 (54.6 %)  30 (27.8 %)  12 (11.1 %)  6 (5.6 %)  1 (0.9 %) | 11.351 | **.0229** |

^§^ CMH chi-square test;

*p*-values less than 0.05 are considered significant and highlighted in bold.

CsA OE: cyclosporine ophthalmic emulsion

Table 5. Comparison of visual fatigue score between 0.05% cyclosporine ophthalmic emulsion (CsA OE) group and vehicle group at different time points during the study in the full analysis set

|  | 0.05%  CsA OE  (N=119） | Vehicle  (N=115) | Statistics^§^  (CMH) | *p* value |
| --- | --- | --- | --- | --- |
| Baseline n (%)  0 - normal  1 - mild  2 - moderate  3 - severe  4 - extremely severe | 14 (11.8 %)  35 (29.4 %)  29 (24.4 %)  28 (23.5 %)  13 (10.9 %) | 11(9.6%)  34 (29.6 %)  32 (27.8 %)  20 (17.4 %)  18 (15.7 %) | 2.5831 | .6298 |
| Day 7 n (%)  0 - normal  1 - mild  2 - moderate  3 - severe  4 - extremely severe | 24 (20.2 %)  44 (37.0 %)  24 (20.2 %)  23 (19.3 %)  4 (3.4 %) | 19 (16.7 %)  38 (33.3 %)  23 (20.2 %)  23 (20.2 %) 11(9.6%) | 4.185 | .3816 |
| Day 28 n (%)  0 - normal  1 - mild  2 - moderate  3 - severe  4 - extremely severe | 38 (33.0 %)  43 (37.4 %)  25 (21.7 %)  8 (7.0 %)  1 (0.9 %) | 23 (20.5 %)  40 (35.7 %)  27 (24.1 %)  16 (14.3 %) 6(5.4%) | 10.03 | **.0399** |
| Day 56 n (%)  0 - normal  1 - mild  2 - moderate  3 - severe  4 - extremely severe | 46 (41.1 %)  45 (40.2 %)  17 (15.2 %)  3 (2.7 %)  1 (0.9 %) | 26 (24.1 %)  40 (37.0 %)  29 (26.9 %)  11 (10.2 %) 2(1.9%) | 13.754 | **.0081** |
| Day 84 n (%)  0 - normal  1 - mild  2 - moderate  3 - severe  4 - extremely severe | 66 (61.7 %)  29 (27.1 %)  9 (8.4 %)  3 (2.8 %)  0 | 35 (32.4 %)  50 (46.3 %)  17 (15.7 %) 4(3.7%)  2(1.9%) | 19.606 | **<0.001** |

^§^ CMH chi-square test;

*p*-values less than 0.05 are considered significant and highlighted in bold.

CsA OE: cyclosporine ophthalmic emulsion

Table 6. Comparison of itching score between 0.05% cyclosporine ophthalmic emulsion (CsA OE) group and vehicle group at different time points during the study in the full analysis set

|  | 0.05%  CsA OE (N=119） | Vehicle (N=115) | Statistics^§^  (CMH) | *p* value |
| --- | --- | --- | --- | --- |
| Baseline n (%)  0 - normal  1 - mild  2 - moderate  3 - severe  4 - extremely severe | 30 (25.2 %)  51 (42.9 %)  20 (16.8 %)  13 (10.9 %)  5 (4.2 %) | 33 (28.7 %)  45 (39.1 %)  22 (19.1 %) 9(7.8%)  6(5.2%) | 1.3575 | .8516 |
| Day 7 n (%)  0 - normal  1 - mild  2 - moderate  3 - severe  4 - extremely severe | 42 (35.3 %)  50 (42.0 %)  18 (15.1 %)  8 (6.7 %)  1 (0.8 %) | 44 (38.6 %)  39 (34.2 %)  20 (17.5 %) 8(7.0%)  3(2.6%) | 2.3948 | .6636 |
| Day 28 n (%)  0 - normal  1 - mild  2 - moderate  3 - severe  4 - extremely severe | 55 (47.8 %)  43 (37.4 %)  16 (13.9 %)  1 (0.9 %)  0 | 58 (51.8 %)  33 (29.5 %)  13 (11.6 %) 6(5.4%)  2(1.8%) | 7.2069 | .1253 |
| Day 56 n (%)  0 - normal  1 - mild  2 - moderate  3 - severe  4 - extremely severe | 65 (58.0 %)  39 (34.8 %)  7 (6.3 %)  1 (0.9 %)  0 | 57 (52.8 %)  32 (29.6 %)  13 (12.0 %)  6 (5.6%)  0 | 6.486 | .0902 |
| Day 84 n (%)  0 - normal  1 - mild  2 - moderate  3 - severe  4 - extremely severe | 74 (69.2 %)  30 (28.0 %)  2 (1.9 %)  1 (0.9 %)  0 | 66 (61.1 %)  29 (26.9 %)  11 (10.2 %) 1(0.9%)  1(0.9%) | 7.6646 | .1047 |

^§^ CMH chi-square test;

*p*-values less than 0.05 are considered significant and highlighted in bold.

CsA OE: cyclosporine ophthalmic emulsion

Table 7. Comparison of stinging score between 0.05% cyclosporine ophthalmic emulsion (CsA OE) group and vehicle group at different time points during the study in the full analysis set

|  | 0.05%  CsA OE  (N=119） | Vehicle  (N=115) | Statistics^§^  (CMH) | *p* value |
| --- | --- | --- | --- | --- |
| Baseline n (%)  0 - normal  1 - mild  2 - moderate  3 - severe  4 - extremely severe | 34 (28.6 %)  47 (39.5 %)  20 (16.8 %)  12 (10.1 %) 6(5.0%) | 41 (35.7 %)  30 (26.1 %)  23 (20.0 %) 11(9.6%) 10(8.7%) | 5.5687 | .2338 |
| Day 7 n (%)  0 - normal  1 - mild  2 - moderate  3 - severe  4 - extremely severe | 42 (35.3 %)  54 (45.4 %)  14 (11.8 %) 7(5.9%)  2(1.7%) | 51 (44.7 %)  31 (27.2 %)  15 (13.2 %) 11(9.6%)  6(5.3%) | 9.8726 | **.0426** |
| Day 28 n (%)  0 - normal  1 - mild  2 - moderate  3 - severe  4 - extremely severe | 47 (40.9 %)  54 (47.0 %)  12 (10.4 %) 2(1.7%)  0 | 57 (50.9 %)  32 (28.6 %)  13 (11.6 %) 9(8.0%)  1(0.9%) | 11.993 | **.0174** |
| Day 56 n (%)  0 - normal  1 - mild  2 - moderate  3 - severe  4 - extremely severe | 60 (53.6 %)  42 (37.5 %) 9(8.0%)  1(0.9%)  0 | 67 (62.0 %)  25 (23.1 %) 10(9.3%)  5(4.6%)  1(0.9%) | 8.3106 | .0808 |
| Day 84 n (%)  0 - normal  1 - mild  2 - moderate  3 - severe  4 - extremely severe | 78 (72.9 %)  25 (23.4 %) 4(3.7%)  0  0 | 65 (60.2 %)  28 (25.9 %) 9(8.3%)  5(4.6%)  1(0.9%) | 9.2271 | .0557 |

^§^ CMH chi-square test;

*p*-values less than 0.05 are considered significant and highlighted in bold.

CsA OE: cyclosporine ophthalmic emulsion

Table 8. Comparison of photophobia score between 0.05% cyclosporine ophthalmic emulsion (CsA OE) group and vehicle group at different time points during the study in the full analysis set

|  | 0.05%  CsA OE  (N=119） | Vehicle  (N=115) | Statistics^§^  (CMH) | *p* value |
| --- | --- | --- | --- | --- |
| Baseline n (%)  0 - normal  1 - mild  2 - moderate  3 - severe  4 - extremely severe | 26 (21.8 %)  41 (34.5 %)  25 (21.0 %)  12 (10.1 %)  15 (12.6 %) | 28 (24.3 %)  40 (34.8 %)  18 (15.7 %)  17 (14.8 %)  12 (10.4 %) | 2.3436 | .6728 |
| Day 7 n (%)  0 - normal  1 - mild  2 - moderate  3 - severe  4 - extremely severe | 34 (28.6 %)  48 (40.3 %)  19 (16.0 %)  13 (10.9 %)  5 (4.2%) | 33 (28.9 %)  44 (38.6 %)  13 (11.4 %)  16 (14.0 %)  8(7.0%) | 2.2007 | .6989 |
| Day 28 n (%)  0 - normal  1 - mild  2 - moderate  3 - severe  4 - extremely severe | 43 (37.4 %)  49 (42.6 %)  14 (12.2 %)  9(7.8%)  0 | 39 (34.8 %)  44 (39.3 %)  14 (12.5 %)  11(9.8%)  4(3.6%) | 4.6047 | .3303 |
| Day 56 n (%)  0 - normal  1 - mild  2 - moderate  3 - severe  4 - extremely severe | 48 (42.9 %)  51 (45.5 %)  13 (11.6 %)  0  0 | 38 (35.2 %)  44 (40.7 %)  18 (16.7 %)  7(6.5%)  1(0.9%) | 10.368 | **.0347** |
| Day 84 n (%)  0 - normal  1 - mild  2 - moderate  3 - severe  4 - extremely severe | 60 (56.1 %)  41 (38.3 %) 6(5.6%)  0  0 | 49 (45.4 %)  40 (37.0 %)  13 (12.0 %)  5(4.6%)  1(0.9%) | 9.6518 | **.0467** |

^§^ CMH chi-square test;

*p*-values less than 0.05 are considered significant and highlighted in bold.

CsA OE: cyclosporine ophthalmic emulsion

Table 9. Comparison of blurred vision score between 0.05% cyclosporine ophthalmic emulsion (CsA OE) group and vehicle group at different time points during the study in the full analysis set

|  | 0.05%  CsA OE  (N=119） | Vehicle  (N=115) | Statistics^§^  (CMH) | *p* value |
| --- | --- | --- | --- | --- |
| Baseline n (%)  0 - normal  1 - mild  2 - moderate  3 - severe  4 - extremely severe | 41 (34.5 %)  40 (33.6 %)  17 (14.3 %)  14 (11.8 %) 7(5.9%) | 32 (27.8 %)  48 (41.7 %)  18 (15.7 %)  9 (7.8%)  8 (7.0%) | 2.9389 | .5681 |
| Day 7 n (%)  0 - normal  1 - mild  2 - moderate  3 - severe  4 - extremely severe | 54 (45.4 %)  37 (31.1 %)  15 (12.6 %)  11 (9.2%)  2 (1.7%) | 45 (39.5 %)  44 (38.6 %)  13 (11.4 %)  7 (6.1%)  5 (4.4%) | 3.6194 | .46 |
| Day 28 n (%)  0 - normal  1 - mild  2 - moderate  3 - severe  4 - extremely severe | 58 (50.4 %)  37 (32.2 %)  17 (14.8 %)  2 (1.7%)  1 (0.9%) | 50 (44.6 %)  42 (37.5 %)  10 (8.9%)  6 (5.4%)  4 (3.6%) | 6.4568 | .1675 |
| Day 56 n (%)  0 - normal  1 - mild  2 - moderate  3 - severe  4 - extremely severe | 67 (59.8 %)  33 (29.5 %) 11(9.8%)  1(0.9%)  0 | 60 (55.6 %)  30 (27.8 %)  10 (9.3%)  7 (6.5%)  1(0.9%) | 5.9783 | .2008 |
| Day 84 n (%)  0 - normal  1 - mild  2 - moderate  3 - severe  4 - extremely severe | 74 (69.2 %)  27 (25.2 %) 6(5.6%)  0  0 | 64 (59.3 %)  34 (31.5 %) 5(4.6%)  4(3.7%)  1(0.9%) | 6.5836 | .1596 |

^§^ CMH chi-square test;

*p*-values less than 0.05 are considered significant and highlighted in bold.

CsA OE: cyclosporine ophthalmic emulsion

Table 10. Comparison of eye redness score between 0.05% cyclosporine ophthalmic emulsion (CsA OE) group and vehicle group at different time points during the study in the full analysis set

|  | 0.05%  CsA OE  (N=119） | Vehicle  (N=115) | Statistics^§^  (CMH) | *p* value |
| --- | --- | --- | --- | --- |
| Baseline n (%)  0 - normal  1 - mild  2 - moderate  3 - severe  4 - extremely severe | 33 (27.7 %)  61 (51.3 %)  18 (15.1 %)  6 (5.0 %)  1 (0.8 %) | 36 (31.3 %)  54 (47.0 %)  16 (13.9 %)  8 (7.0 %)  1 (0.9 %) | .888 | .9263 |
| Day 7 n (%)  0 - normal  1 - mild  2 - moderate  3 - severe  4 - extremely severe | 46 (38.7 %)  57 (47.9 %)  12 (10.1 %)  4 (3.4 %)  0 | 41 (35.7 %)  51 (44.3 %)  15 (13.0 %)  7 (6.1 %)  1 (0.9 %) | 2.6931 | 0.6104 |
| Day 28 n (%)  0 - normal  1 - mild  2 - moderate  3 - severe  4 - extremely severe | 59 (49.6 %)  48 (40.3 %)  11 (9.2 %)  1 (0.8 %)  0 | 42 (36.5 %)  59 (51.3 %)  10 (8.7 %)  3 (2.6 %)  1（0.9%） | 5.9477 | 0.2031 |
| Day 56 n (%)  0 - normal  1 - mild  2 - moderate  3 - severe  4 - extremely severe | 73 (61.3 %)  38 (31.9 %)  8 (6.7 %)  0  0 | 53 (46.1 %)  51 (44.3 %)  9(7.8%)  2 (1.7 %)  0 | 7.0358 | 0.0708 |
| Day 84 n (%)  0 - normal  1 - mild  2 - moderate  3 - severe  4 - extremely severe | 81 (68.1 %)  34 (28.6 %)  4 (3.4 %)  0  0 | 61 (53.0 %)  46 (40.0 %)  6(5.2%)  2(1.7%)  0 | 6.9209 | .0745 |

^§^ CMH chi-square test;

*p*-values less than 0.05 are considered significant and highlighted in bold.

CsA OE: cyclosporine ophthalmic emulsion

Table 11. Comparison of Schirmer I test between 0.05% cyclosporine ophthalmic emulsion (CsA OE) group and vehicle group at different time points during the study in the full analysis set

|  | 0.05%  CsA OE  (N=119） | Vehicle (N=115) | Statistics^§^  (CMH) | *p* value |
| --- | --- | --- | --- | --- |
| Baseline n (%)  0 - normal  1 - mild  2 - moderate  3 - severe  4 - extremely severe | 5(4.2%)  12 (10.1 %)  51 (42.9 %)  28 (23.5 %)  23 (19.3 %) | 4(3.5%)  12 (10.4 %)  45 (39.1 %)  41 (35.7 %)  13 (11.3 %) | 5.6223 | .2292 |
| Day 7 n (%)  0 - normal  1 - mild  2 - moderate  3 - severe  4 - extremely severe | 10(8.4%)  25 (21.0 %)  47 (39.5 %)  25 (21.0 %)  12 (10.1 %) | 4 (3.5 %)  17 (14.9 %)  48 (42.1 %)  32 (28.1 %)  13 (11.4 %) | 4.8793 | .2999 |
| Day 28 n (%)  0 - normal  1 - mild  2 - moderate  3 - severe  4 - extremely severe | 17 (14.8 %)  34 (29.6 %)  38 (33.0 %)  22 (19.1 %)  4 (3.5 %) | 7 (6.3 %)  17 (15.2 %)  45 (40.2 %)  32 (28.6 %)  11 (9.8 %) | 15.437 | **.0039** |
| Day 56 n (%)  0 - normal  1 - mild  2 - moderate  3 - severe  4 - extremely severe | 18 (16.2 %)  42 (37.8 %)  36 (32.4 %)  14 (12.6 %)  1 (0.9 %) | 7 (6.5 %)  17 (15.7 %)  50 (46.3 %)  28 (25.9 %)  6 (5.6 %) | 25.796 | **<.001** |
| Day 84 n (%)  0 - normal  1 - mild  2 - moderate  3 - severe  4 - extremely severe | 25 (23.4 %)  39 (36.4 %)  29 (27.1 %)  14 (13.1 %)  0 | 7 (6.5 %)  18 (16.7 %)  52 (48.1 %)  25 (23.1 %)  6 (5.6 %) | 33.336 | **<.001** |

^§^ CMH chi-square test;

Schirmer I test was scored as follows: 0, > 10mm/5min; 1, ≤10 and >5 mm/5min; 2, ≤5 and >2 mm/5min; 3, ≤2 and >0 mm/5min; 4, 0 mm/5min.

*p*-values less than 0.05 are considered significant and highlighted in bold.

CsA OE: cyclosporine ophthalmic emulsion

Table 12. Comparison of tear film break-up time (TBUT) between 0.05% cyclosporine ophthalmic emulsion (CsA OE) group and vehicle group at different time points during the study in the full analysis set

|  | 0.05%  CsA OE  (N=119） | Vehicle  (N=115) | Statistics^§^  (CMH) | *p* value |
| --- | --- | --- | --- | --- |
| Baseline n (%)  0 - normal  1 - mild  2 - moderate  3 - severe  4 - extremely severe | 0  10(8.4%)  33 (27.7 %)  61 (51.3 %)  15 (12.6 %) | 0  9 (7.8 %)  31 (27.0 %)  58 (50.4 %)  17 (14.8 %) | 0.2464 | .9698 |
| Day 7 n (%)  0 - normal  1 - mild  2 - moderate  3 - severe  4 - extremely severe | 1 (0.8 %)  13 (10.9 %)  49 (41.2 %)  47 (39.5 %)  9 (7.6 %) | 1 (0.9 %)  9 (7.9 %)  37 (32.5 %)  52 (45.6 %)  15 (13.2 %) | 4.0314 | .4018 |
| Day 28 n (%)  0 - normal  1 - mild  2 - moderate  3 - severe  4 - extremely severe | 0  24 (20.9 %)  60 (52.2 %)  25 (21.7 %)  6 (5.2 %) | 1 (0.9 %)  15 (13.4 %)  32 (28.6 %)  54 (48.2 %)  10 (8.9 %) | 23.106 | **<0.001** |
| Day 56 n (%)  0 - normal  1 - mild  2 - moderate  3 - severe  4 - extremely severe | 0  42 (37.5 %)  51 (45.5 %)  15 (13.4 %)  4 (3.6 %) | 0  14 (13.0 %)  51 (47.2 %)  34 (31.5 %)  9 (8.3 %) | 23.12 | **<0.001** |
| Day 84 n (%)  0 - normal  1 - mild  2 - moderate  3 - severe  4 - extremely severe | 10 (9.3%)  46 (43.0 %)  37 (34.6 %)  11 (10.3 %)  3 (2.8 %) | 0  18 (16.7 %)  47 (43.5 %)  36 (33.3 %)  7 (6.5 %) | 38.156 | **<0.001** |

^§^ CMH chi-square test;

TBUT was scored as follows: 0, > 10s; 1, ≤10 and >5s; 2, ≤5 and >2s; 3, ≤2 and >0s; 4, 0s.

*p*-values less than 0.05 are considered significant and highlighted in bold.

CsA OE: cyclosporine ophthalmic emulsion

Table 13. Comparison of corneal fluorescein and conjunctival lissamine green staining (Oxford scheme) between 0.05% cyclosporine ophthalmic emulsion (CsA OE) group and vehicle group at different time points during the study in the full analysis set

|  | 0.05%  CsA OE  (N=119） | Vehicle  (N=115) | Statistics*  (F) | *p* value |
| --- | --- | --- | --- | --- |
| Baseline n (%)  n (n miss)  Mean + SD  Median  P25 - P75  Min - Max | 119(0)  7.75 + 2.94  7.00  6.00 ~ 9.00  2.00 ~ 15.00 | 115(0)  7.47 + 3.22  7.00  5.00 ~ 10.00  1.00 ~ 15.00 | 0.478 | .49 |
| Day 7 n (%)  n (n miss)  Mean + SD  Median  P25 - P75  Min - Max | 119(0)  6.20 + 3.10  6.00  4.00 ~ 8.00  1.00 ~ 15.00 | 114(1)  6.77 + 3.16  6.00  4.00 ~ 9.00  0.00 ~ 15.00 | 1.9325 | .1658 |
| Day 28 n (%)  n (n miss)  Mean + SD  Median  P25 - P75  Min - Max | 115(4)  4.89 + 2.79  5.00  3.00 ~ 6.00  0.00 ~ 14.00 | 112(3)  5.96 + 3.38  6.00  4.00 ~ 8.00  0.00 ~ 15.00 | 6.8745 | **.0093** |
| Day 56 n (%)  n (n miss)  Mean + SD  Median  P25 - P75  Min - Max | 112(7)  3.63 + 2.39  3.00  2.00 ~ 5.00  0.00 ~ 14.00 | 108(7)  5.09 + 3.48  4.50  3.00 ~ 7.00  0.00 ~ 15.00 | 13.193 | **<.001** |
| Day 84 n (%)  n (n miss)  Mean + SD  Median  P25 - P75  Min - Max | 107(12)  2.50 + 2.17  2.00  1.00 ~ 4.00  0.00 ~ 10.00 | 108(7)  4.59 + 3.49  4.00  2.00 ~ 6.00  0.00 ~ 15.00 | 27.622 | **<.001** |

^*^ Student’s t-test or Wilcoxon rank sum test;

*p*-values less than 0.05 are considered significant and highlighted in bold.

CsA OE: cyclosporine ophthalmic emulsion
